# Supplementary material for: Pyruvate Dehydrogenase Kinase 1 inhibition mediated oxidative phosphorylation enhancement in cartilage promotes osteoarthritis progression
Source: BMC Musculoskelet Disord. 2023 Jul 20;24:597. doi: 10.1186/s12891-023-06585-6 (PMC10357736; doi:10.1186/s12891-023-06585-6)
Supplement: Supplementary file 1 — Additional file 1. [file 12891_2023_6585_MOESM1_ESM.zip › MF.Gsea.1653627316242/GOMF_L_ASCORBIC_ACID_BINDING.html]

Details for gene set GOMF\_L\_ASCORBIC\_ACID\_BINDING[GSEA]

|  || Dataset | 55457\_collapsed\_to\_symbols.cls.cls #OA\_versus\_normal.cls.cls #OA\_versus\_normal\_repos |
| Phenotype | cls.cls#OA\_versus\_normal\_repos |
| Upregulated in class | OA |
| GeneSet | GOMF\_L\_ASCORBIC\_ACID\_BINDING |
| Enrichment Score (ES) | 0.4451798 |
| Normalized Enrichment Score (NES) | 1.3505015 |
| Nominal p-value | 0.122119814 |
| FDR q-value | 0.3531307 |
| FWER p-Value | 1.0 |
Table: GSEA Results Summary

  

Fig 1: Enrichment plot: GOMF\_L\_ASCORBIC\_ACID\_BINDING      
 Profile of the Running ES Score & Positions of GeneSet Members on the Rank Ordered List

  

| SYMBOL | TITLE | RANK IN GENE LIST | RANK METRIC SCORE | RUNNING ES | CORE ENRICHMENT || 1 | P4HA2 | prolyl 4-hydroxylase subunit alpha 2 [Source:HGNC Symbol;Acc:HGNC:8547] | 258 | 0.779 | 0.1410 | Yes |
| 2 | P4HA1 | prolyl 4-hydroxylase subunit alpha 1 [Source:HGNC Symbol;Acc:HGNC:8546] | 833 | 0.560 | 0.2121 | Yes |
| 3 | OGFOD3 | 2-oxoglutarate and iron dependent oxygenase domain containing 3 [Source:HGNC Symbol;Acc:HGNC:26174] | 938 | 0.531 | 0.3137 | Yes |
| 4 | OGFOD1 | 2-oxoglutarate and iron dependent oxygenase domain containing 1 [Source:HGNC Symbol;Acc:HGNC:25585] | 1360 | 0.449 | 0.3737 | Yes |
| 5 | PHYH | phytanoyl-CoA 2-hydroxylase [Source:HGNC Symbol;Acc:HGNC:8940] | 2397 | 0.315 | 0.3583 | Yes |
| 6 | P3H1 | prolyl 3-hydroxylase 1 [Source:HGNC Symbol;Acc:HGNC:19316] | 2853 | 0.265 | 0.3777 | Yes |
| 7 | PLOD1 | "procollagen-lysine,2-oxoglutarate 5-dioxygenase 1 [Source:HGNC Symbol;Acc:HGNC:9081]" | 3155 | 0.235 | 0.4029 | Yes |
| 8 | PLOD3 | "procollagen-lysine,2-oxoglutarate 5-dioxygenase 3 [Source:HGNC Symbol;Acc:HGNC:9083]" | 3614 | 0.192 | 0.4070 | Yes |
| 9 | OGFOD2 | 2-oxoglutarate and iron dependent oxygenase domain containing 2 [Source:HGNC Symbol;Acc:HGNC:25823] | 3632 | 0.191 | 0.4452 | Yes |
| 10 | PAM | peptidylglycine alpha-amidating monooxygenase [Source:HGNC Symbol;Acc:HGNC:8596] | 4373 | 0.123 | 0.4131 | No |
| 11 | DBH | dopamine beta-hydroxylase [Source:HGNC Symbol;Acc:HGNC:2689] | 6133 | -0.015 | 0.2797 | No |
| 12 | EGLN1 | egl-9 family hypoxia inducible factor 1 [Source:HGNC Symbol;Acc:HGNC:1232] | 6364 | -0.032 | 0.2686 | No |
| 13 | EGLN3 | egl-9 family hypoxia inducible factor 3 [Source:HGNC Symbol;Acc:HGNC:14661] | 8965 | -0.229 | 0.1142 | No |
| 14 | P3H2 | prolyl 3-hydroxylase 2 [Source:HGNC Symbol;Acc:HGNC:19317] | 9254 | -0.252 | 0.1439 | No |
| 15 | PLOD2 | "procollagen-lysine,2-oxoglutarate 5-dioxygenase 2 [Source:HGNC Symbol;Acc:HGNC:9082]" | 10086 | -0.330 | 0.1476 | No |
| 16 | EGLN2 | egl-9 family hypoxia inducible factor 2 [Source:HGNC Symbol;Acc:HGNC:14660] | 10212 | -0.342 | 0.2086 | No |
Table: GSEA details [plain text format]

  

Fig 2: GOMF\_L\_ASCORBIC\_ACID\_BINDING      
 Blue-Pink O' Gram in the Space of the Analyzed GeneSet

  

Fig 3: GOMF\_L\_ASCORBIC\_ACID\_BINDING: Random ES distribution      
 Gene set null distribution of ES for **GOMF\_L\_ASCORBIC\_ACID\_BINDING**
